# The European Bioinformatics Institute in 2016: Data growth and integration

**DOI:** 10.1093/nar/gkv1352

**Published:** 2015-12-15

**Authors:** Charles E. Cook, Mary Todd Bergman, Robert D. Finn, Guy Cochrane, Ewan Birney, Rolf Apweiler

**Affiliations:** European Molecular Biology Laboratory, European Bioinformatics Institute (EMBL-EBI), Wellcome Genome Campus, Hinxton, Cambridge CB10 1SD, UK

## Abstract

New technologies are revolutionising biological research and its applications by making it easier and cheaper to generate ever-greater volumes and types of data. In response, the services and infrastructure of the European Bioinformatics Institute (EMBL-EBI, www.ebi.ac.uk) are continually expanding: total disk capacity increases significantly every year to keep pace with demand (75 petabytes as of December 2015), and interoperability between resources remains a strategic priority. Since 2014 we have launched two new resources: the European Variation Archive for genetic variation data and EMPIAR for two-dimensional electron microscopy data, as well as a Resource Description Framework platform. We also launched the Embassy Cloud service, which allows users to run large analyses in a virtual environment next to EMBL-EBI's vast public data resources.

## INTRODUCTION

EMBL-EBI data resources are freely available and cover the entire range of biological sciences, from raw DNA sequences to curated proteins, chemicals, structures, systems, pathways, ontologies and literature (1). The institute expands these offerings continually to reflect technological changes that lead to the generation of new data types. We also adapt our services to accommodate the exponential growth of biological data enabled by advances in molecular technologies. We have a mandate to provide freely available data and bioinformatics services to the scientific community, and to make public data resources accessible through user-centred design. Accordingly, we make biological data discoverable though web browsers, application programming interfaces (APIs), scalable search technology and extensive cross-referencing between databases. In this update we describe the tremendous growth in biological data stored in the public archives, illustrate the extensive cross-references we maintain to enhance usability and discoverability and describe a selection of developments in our services since 2014.

## DATA GROWTH AND INTERCONNECTIVITY

Biology is in the midst of a revolution: new technologies are making it easier and cheaper to undertake experiments that generate vast quantities of data, which in turn requires more biologists to work computationally and more data to be shared in the public archives. Recent projections, and our own observations, suggest that biological data volumes will soon rival those produced by astronomical observation (2). Most funders now require deposition of data in publicly accessible data repositories, and much of the data generated through these new technologies is deposited at EMBL-EBI. There are significant challenges in processing, storing and analysing these data and many opportunities unlocked by integrating them in ways that encourage the generation of new knowledge.

**Figure 1.**
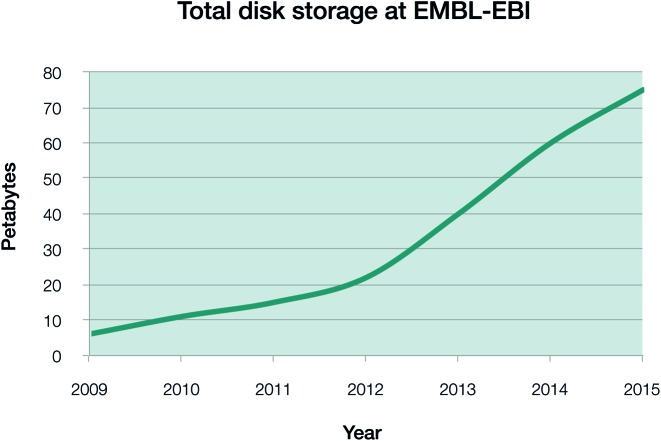
Installed (2008–2015) storage at EMBL-EBI. These figures include all installed storage, counting multiple backups for all data resources as well as unused storage to handle submissions in the immediate future. The actual total volume of a single copy of all data resources is roughly 30% of total installed storage capacity. Figures are for end-of-year; 2015 figure is estimated based on installed capacity in October 2015.

**Figure 2.**
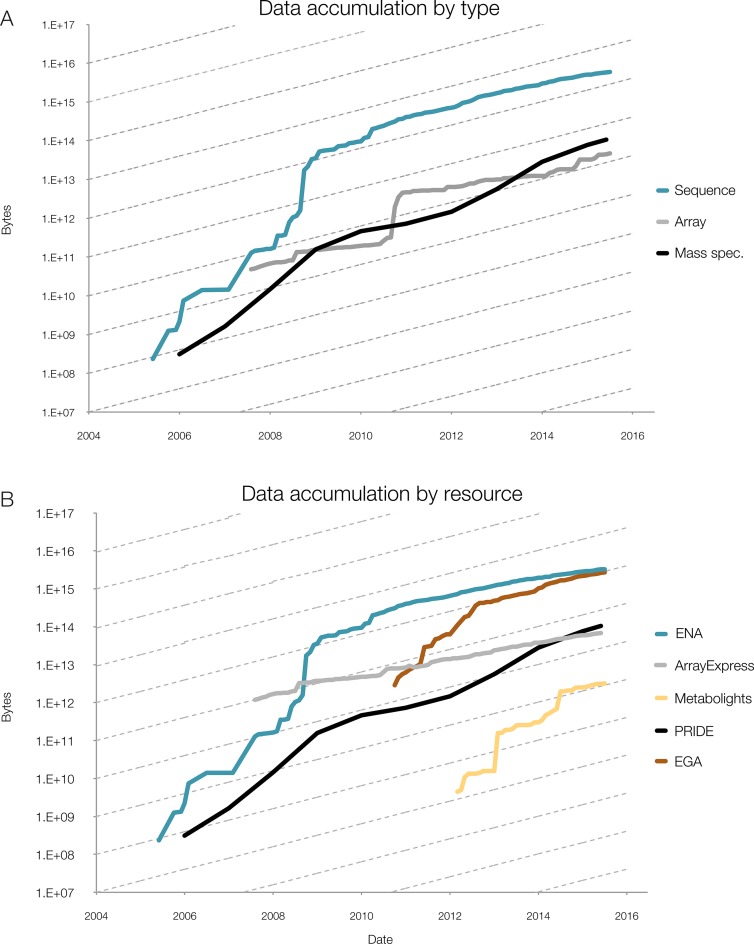
(**A**) Data accumulation at EMBL-EBI by data type, for example mass spectrometry (MS); (**B**) Data accumulation by dedicated resource, for example PRIDE. The y-axis is log-scale, with the slope of the dashed lines indicating a 12-month doubling time. Continued data growth is seen in all types of data at EMBL-EBI and all data resources. In all data resources shown here, growth rates are predicted to continue increasing, with notable sustained exponential growth in PRIDE, the European Genome-phenome Archive (EGA) and MetaboLights: all have doubling times of around 12 months. All three contributing platforms show rates that are increasing over time, with data growing exponentially with around a 12-month doubling time.

Data storage capacity (Figure 1) has grown in a linear fashion, while nucleotide and proteomics data generation has grown exponentially (Figure 2). This situation presents substantial challenges to keeping these data in the public domain, and is not sustainable in the long term. Compression techniques such as CRAM (3,4) resolve one important issue: handling nucleotide data on a very large scale, so developing novel compression methods is an important part of the institute's work. Beyond storage, our central tasks involve building tools that make it easier for researchers to interpret the data, enriching existing resources, creating new ones and integrating them to maximise their utility.

There are both infrastructural and organisational challenges inherent to managing resources that are growing exponentially. We are continually installing new storage and computational hardware to accommodate newly submitted data and to ensure users can access them: larger data volumes can lead to searches becoming increasingly time consuming. In response, the EBI Search was developed as a scalable system that can satisfy user search queries regardless of the volume of data being searched (5). In addition, EMBL-EBI is engaging other institutions across Europe through ELIXIR (www.elixir-europe.org), the European research infrastructure for life sciences, to coordinate and implement distributed solutions to challenges of storing and curating biological data.

**Figure 3.**
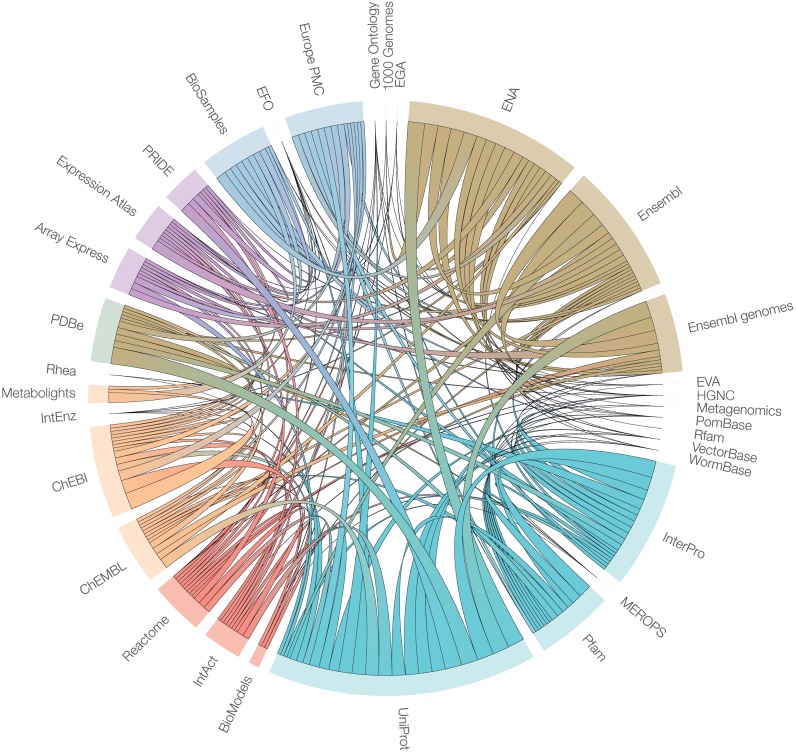
Representation of the internal interactions between different databases and resources at the EMBL-EBI, as determined by the exchange of data. All resources are placed on the circumference of the circle, with each resource represented by an arc proportional to the total number of interactions. The width of each internal arc, which transects the circle and connects two different resources, is weighted according to the number of different data types that are exchange between the two resources at the ends of the arc. The colouring of the internal arcs does not reflect the direction of data exchange. The graphic was generated using the D3 JavaScript library (http://d3js.org) and the data, gathered as part of an external review, were accurate at the time of acquisition (Jan 2015).

EMBL-EBI data resources mirror living functions, so their integration enables progress towards virtual systems that simulate the continual interactions between cellular components. Our goal is to show users all of the most relevant and useful information for their research, whether it is a gene, gene expression profile, protein sequence, molecular structure, chemical compound, pathway, patent or literature reference. The EBI Search and the Web Services framework (6) make extensive use of cross-references to return relevant results to our users across different resources (Figure 3), recognising that substantial interactions between databases enhance the value and experience to the end user.

## NEW AND UPDATED DATA RESOURCES

### Genes, genomes and variation

The European Nucleotide Archive (7) and EMBL-EBI's Metagenomics service (8) offer different ways to access data from the *Tara* Oceans expeditions (9), which produced the largest and most richly detailed collection of data about plankton in the world's oceans. Detailed updates of these services, Ensembl (10), Ensembl Genomes (11) and PhytoPath (12) are provided elsewhere in this issue.

High-profile genetic variation datasets have been made publicly available by groups such as the 1000 Genomes Project (13), the Exome Aggregation Consortium (ExAC), deCODE (14) and UK10K (15). These datasets are often prepared using independent methodologies or processing pipelines, and can be accessed only through custom websites or FTP servers. EMBL-EBI launched the European Variation Archive (EVA, http://www.ebi.ac.uk/eva) in October of 2014 to provide a single access point for submissions, archiving and access to high-resolution variation data of all types. The EVA gathers, normalises and annotates (via standardised pipelines) the variants from externally hosted datasets as well as those submitted directly to EMBL-EBI.

At its launch, the EVA contained variants from large-scale efforts including the 1000 Genomes Project, Exome Variant Server and Genome of the Netherlands Project and UK10K. Agriculturally relevant species including sheep, cow, maize and tomato were added soon after launch, extending the EVA's utility and demonstrating the flexibility of the technology. As of October 2015, the EVA describes data from more than 40 studies, representing 35 species and describing about 400 million unique alleles from more than 150 000 samples. These data are available through the EVA browser, which accommodates both study-centric and global queries, filtering on any combination of species, gene, variant consequence or protein substitution score(s). The EVA provides a comprehensive RESTful Web Service to allow programmatic access, which facilitates integration with other resources such as ArrayExpress and UniProt.

In collaboration with ClinVar (16) at the National Center for Biotechnology Information (NCBI), EVA offers a clinically focused resource that details the largest public collection of variant-to-phenotype relationships available worldwide. Each of the 135 000 variants in this dataset is associated with at least one phenotype and a clinical classification from the American College of Medical Genetics and Genomics ACMG guidelines (17). EVA integrates all clinical variants with other clinically focused datasets such as the Leiden Open Variation Database (18) and the Online Mendelian Inheritance in Man (OMIM) catalogue (19).

### RNA

RNAcentral (http://rnacentral.org) (20) is a database of non-coding RNA sequences that serves as a single entry point for information stored in 36 specialised RNA databases, including Rfam (21), miRBase (22) and Vega (23). RNAcentral assigns unique identifiers to each distinct sequence and lets users search, view and download the data, or navigate to the specialised databases for more detailed annotations. Since its release in September 2014, RNAcentral has introduced new features, including species-specific sequence identifiers, which are now used for non-coding RNA curation by the Gene Ontology Consortium. It has also introduced a sequence search powered by nhmmer (24) and imported non-coding RNA sequences from the Protein Data Bank in Europe (PDBe (25)), snOPY (26), the Saccharomyces Genome Database (27), The Arabidopsis Information Resource (28) and WormBase (29).

Detailed updates of the Gene Expression Atlas (30) and the PRIDE (31) resource for proteomics data are provided elsewhere in this issue.

### Proteins

UniProt, the Universal Protein resource, is a collaboration between the European Bioinformatics Institute (EMBL-EBI), the SIB Swiss Institute of Bioinformatics and the Protein Information Resource (PIR). Since the last update in 2015 (32), a significant change to UniProt has been the removal, using newly developed procedures, of highly redundant proteomes generated by sequencing of hundreds or even thousands of near-to-identical bacterial isolates. UniProtKB release 2015_04 first implemented these changes, reducing the total number of sequences in UniProtKB from 92 million to 47 million, and the process is now ongoing with each release. Redundant proteomes removed from UniProtKB are still available in UniParc. This change increases the scalability of the UniProt pipelines and the many other bioinformatics pipelines worldwide that rely on UniProt data. UniProt retains the ability to promote proteomes back to UniProtKB/TrEMBL if requested by the community. For other news about our achievements this year see http://www.uniprot.org/help/?fil=section:news.

In 2015 the Enzyme Portal was fully integrated with UniProt and the EBI Search, integrating information from UniProtKB, the Protein Data Bank in Europe (PDBe), Rhea, Reactome, IntEnz, ChEBI and ChEMBL. The service provides a concise summary of protein sequences and their function, small-molecule chemistry, biochemical pathways, drug-like compounds, catalytic activity, taxonomy information and cross-references to the underlying data resources.

The HMMER algorithm, updated in 2015, was launched in a dedicated website at EMBL-EBI. HMMER (www.ebi.ac.uk/Tools/HMMER) provides sophisticated probability models through a simple interface that enables very fast searches of large protein sequence databases, using a single sequence, multiple sequence alignment or profile hidden Markov model as a query. Filters for taxonomy and domain architecture simplify the interpretation of results. HMMER is incorporated into the Pfam (33) and InterPro (34) data services, making it simpler for researchers to identify sequence relationships deep in evolutionary time. Detailed updates for the Pfam and MEROPS (35) databases are provided elsewhere in this issue.

### Macromolecular structures

Cryo-electron microscopy (cryo-EM), an important structural biological technique for the elucidation of the three-dimensional (3D) structure of biological macromolecules, complexes and assemblies, is emerging as the preferred imaging technique for structural biology. Issues with image resolution have largely been resolved since 2013 thanks to new detector technology, better microscopes and improved image processing techniques, though methods for validation are not yet fully mature. The Electron Microscopy Data Bank (EMDB) was created in 2002 in response to growing community need for the archiving of final 3D reconstructions resulting from EM experiments and now offers over 3000 structures that can be examined, validated, compared with other structures and used as a reference. There has been increasing demand for the archive to also offer raw image data in the interests of referencing the data before image processing and 3D reconstruction and improving validation methods.

In 2014, PDBe established the Electron Microscopy Pilot Image Archive (EMPIAR, http://pdbe.org/empiar) for raw two-dimensional image data related to EMDB entries. EMPIAR now holds over 30 datasets, several of which are over 1 terabyte (TB) in size. Users around the world download EMPIAR data on a regular basis for validation, methods development, training and re-processing. It is the source of raw data for the EMDataBank Map Validation Challenge.

A detailed update of PDBe (36) is provided elsewhere in this issue.

### Chemical biology

The MetaboLights (www.ebi.ac.uk/metabolights/) open-access repository for primary metabolomics data is now recommended by journals including *Nature Scientific Data*, the *EMBO journal*, *Metabolomics* and the PLOS journals. MetaboLights supports the submission of metadata and primary raw data through an upload and approval process that allows both submitters and curators to check the status of an on-going study at any time. Based on the ISA framework, it provides a means to capture Metabolomics Standards Initiative (MSI)-compliant metabolomics metadata and raw experimental data. Each submission receives a stable and unique identifier and a reference layer collects structural and chemical information, nuclear magnetic resonance and MS spectra, target species, metabolic pathways and reactions for the associate metabolites within the metabolomics studies.

The metabolomeXchange system (http://metabolomexchange.org/site/), developed by the EMBL-EBI-coordinated Coordination of Standards in Metabolomics (COSMOS) project, enables users to query metabolomics data efficiently and to readily identify interesting and reusable metabolomics datasets (37).

A detailed update of SureChEMBL (38), the collection of chemical data extracted from the patent literature, and of ChEBI (39), the dictionary of molecular entities focused on ‘small’ chemical compounds are provided elsewhere in this issue.

### Pathways and systems

The Reactome pathway browser (http://www.reactome.org) has been updated substantially, with better visualisation and improved tools for searching pathway diagrams. A panel that clearly shows participating molecules and their expression values as well as related pathways, putting molecular reaction information in context. Users can now export pathways as images, including analysis results and other items.

EMBL-EBI is a central member of the Drug Disease Model Resources (DDMoRe (40)) consortium, which launched a new repository for computational models of disease (http://ddmore.eu/model-repository) in 2015. The DDMoRe repository is built on the Pharmacometrics Markup Language (PharmML (41), also led by EMBL-EBI) and features a unique interoperability framework. This allows users to encode their models in a single format that can be converted seamlessly and executed in commonly used software packages, making it easier for researchers to share and reuse models of drug action and disease progression using their own software.

## CROSS-DOMAIN TOOLS AND RESOURCES

### Semantic web

Collaboration and discussion with members of the EMBL-EBI Industry Programme led to the launch in 2013 of the Resource Description Framework (RDF) platform (http://www.ebi.ac.uk/rdf/) (42), which combines EMBL-EBI resources that support semantic web technologies. RDF provides easy links between related but differently structured information, enabling the meaningful and intuitive sharing of molecular data among different applications. There are currently six resources available in the RDF platform: Reactome, BioModels, BioSamples, Expression Atlas, ChEMBL; and the UniProt RDF (sparql.uniprot.org), which is maintained by The SIB-Swiss Institute of Bioinformatics. The RDF platform can be used to query across datasets, for example a query for gene expression data will integrate results from the Expression Atlas with relevant pathway information from Reactome and compound-target information from ChEMBL. The RDF data are available for download or can be queried directly using SPARQL via the open-source Lodestar application (http://www.ebi.ac.uk/fgpt/sw/lodestar/). Lodestar, developed at EMBL-EBI, is used by the National Library of Medicine MeSH linked data browser.

### Embassy Cloud

Large-scale data analyses are becoming the norm in the life sciences, but few organisations have the technical infrastructure in place to carry this work out on a regular basis. The Embassy Cloud, launched in 2013, is an ‘infrastructure as a service’ that enables groups to work in private, secure, virtual-machine-based workspaces hosted within EMBL-EBI's data centres. Embassy Cloud offers users the administrative autonomy to design networks, implement security and manage users within their private workspace, yet is physically next to EMBL-EBI data resources, negating the need to download large data resources. Virtual machines and analysis pipelines in the Embassy Cloud are accessible from anywhere with an Internet connection. As of October 2015, the Embassy Cloud is primarily for external groups that are collaborating with EMBL-EBI teams; however, subject to funding, we aim to expand service to be more widely available.

To enable ELIXIR users to deploy workloads to the Embassy Cloud, we are joining the service to the European Grid Infrastructure (EGI) federation. This will allow ELIXIR users to authenticate with the EGI portal, select an ELIXIR workload and have it deployed to the Embassy without necessitating a dedicated Embassy space or local user.

The Embassy Cloud infrastructure now includes 1200 cores, 11 TB of RAM, 50 TB of solid-state drive (SSD) fast scratch space and 1.2 petabytes of spinning disk storage. In 2015 we moved from VMware vCloud to the OpenStack cloud platform. To help overcome the challenges users face in terms of local expertise and experience in systems administration we plan to share and maintain common virtual machine templates, with documentation and to build a user community for sharing expertise and best practice.

### Training

As our user base has grown and diversified, so has the need to grow and diversify our training offerings. The EMBL-EBI Training Programme provides both online and face-to-face training for molecular life scientists, enabling them to access, analyse and interpret the vast wealth of data managed by EMBL-EBI and its collaborators. In addition to our active series of on-site courses and workshops, we offer introductory and in-depth courses in our Train online e-learning resource (http://www.ebi.ac.uk/training/online), a webinar series and a unified collection of tutorials on the EMBL-EBI YouTube channel. All EMBL-EBI's courses adhere to LifeTrain's agreed principles for course providers (43,44). Our courses are delivered by scientists and engineers whose main roles are research and development, and we use a quality-control mechanism (45) to test the relevance and usability of our courses, ensuring they are well aligned with the data resources they incorporate. To enhance the quality of bioinformatics training worldwide, EMBL-EBI also offers trainer support sessions and, in collaboration with academic and commercial partners, a training toolkit (http://www.on-course.eu/toolkit).

## CONCLUDING REMARKS

EMBL-EBI has a mandate to deliver freely available access to biological data, and to make knowledge accessible to users working in all areas of biology. We continue to deliver a comprehensive range of services in the face of tremendous, exciting changes in the biological sciences, which include exponential increases in data volumes, technological advances that produce new data types and a diversifying scientific and technical workforce. The challenges are not only technical: we also face challenges in serving users in resource-poor geographic locations, and in serving medical and healthcare professionals whose needs are applied rather than research-driven. We are committed to being proactive in understanding the needs of all our users and engaging with new communities and will provide an update of these efforts in future.
